# Transcriptomic Characterization of Phototransduction Genes of the Asian Citrus Psyllid *Diaphorina citri* Kuwayama

**DOI:** 10.3390/insects15120966

**Published:** 2024-12-04

**Authors:** Shao-Ping Chen, Xue-Mei Chu, Mei-Xiang Chi, Jian Zhao, Rong-Zhou Qiu

**Affiliations:** 1 State Key Laboratory of Ecological Pest Control for Fujian and Taiwan Crops, Institute of Plant Protection, Fujian Academy of Agricultural Sciences, Fuzhou 350013, China; fafucsp@163.com (S.-P.C.); 15165546235@163.com (X.-M.C.); cmx895619@163.com (M.-X.C.); zhaojian@faas.cn (J.Z.); 2 Fujian Key Laboratory for Monitoring and Integrated Management of Crop Pests, Fujian Engineering Research Center for Green Pest Management, Fuzhou 350013, China; 3 Fujian Academy of Agricultural Sciences, Fuzhou 350003, China

**Keywords:** Asian citrus psyllid, phototransduction, *LW-opsin*, phototactic behavior, RNA-Seq

## Abstract

Opsin plays a regulatory role in phototaxis of *Diaphorina citri*, functioning as the initial station in the phototransduction cascade. Our study aimed to explore the *D. citri* phototransduction pathway to identify elicitors that may enhance *D. citri* phototaxis in the future. The RNAi technique was employed to inhibit *LW-opsin* gene expression, followed by RNA-Seq analysis to identify phototransduction genes. Finally, RT-qPCR was performed to validate whether genes in the phototransduction pathway were affected by the inhibition of *LW-opsin* expression. A total of 87 genes were identified within the transcriptome as involved in phototransduction based on KEGG functional annotation. Of these, 71 genes were identified as enriched in the phototransduction-fly pathway. These genes encode key proteins in this process, including *Gqα*, *Gqβ*, *Gqγ*, *phospholipase C β* (*PLCβ*), the cation channel *transient receptor potential* (*TRP*), and *TRP-like* (*TRPL*), among others. Moreover, the *LOC103513214* (*Gqβ*) and *LOC103518375* (*ninaC*) genes exhibited reduced expression when *LW-opsin* gene expression was suppressed. Our results provide a basis for further investigation of phototransduction in *D. citri*.

## Simple Summary

Opsin contributes to the phototaxis of Asian citrus psyllid (ACP, *Diaphorina citri*), which is the primary vector of *Candidatus* Liberibacter asiaticus, causing Citrus Huanglongbing (HLB). The objective of this study was to explore the phototransduction pathway of *D. citri* with a view to identifying elicitors that could enhance the phototaxis of *D. citri* in the future. To that end, 87 genes were identified via transcriptome sequencing. Of these genes, 71 were linked to the phototransduction-fly pathway. In addition, when *LW-opsin* gene expression was inhibited, *Gqβ* and *ninaC* were down-regulated. The findings of our study provide a foundation for further research into the phototransduction pathway in ACP, as well as a basis for further investigation into the potential of elicitors to enhance the phototaxis of ACP.

## 1. Introduction

*Diaphorina citri* Kuwayama (Asian citrus psyllid, Hemiptera, Psyllidae) is one of the most significant pests of citrus. It is the primary vector of *Candidatus* Liberibacter asiaticus, the causal agent of Citrus Huanglongbing (HLB) [1,2]. HLB is a destructive disease, often referred to as the “cancer of the citrus industry”, which represents a substantial threat to the global citrus industry and poses a considerable risk to the industry’s sustainability [3,4,5]. The most effective method for controlling HLB is the management of the Asian citrus psyllid through chemical, biological, and physical control measures. Among these, physical control, which leverages the insect’s phototactic behavior, is widely employed in field production due to its high efficacy and environmental compatibility. The Asian citrus psyllid displays positive phototaxis, exhibiting a preference for ultraviolet, blue, and green light [6,7,8]. It has been demonstrated that opsin regulates phototaxis in ACP [8]; however, the underlying molecular mechanism is not fully understood.

Opsin is a member of the G-protein-coupled receptor family, comprising seven transmembrane domains [9,10]. Based on the type of photoreceptor cell, opsins are classified into two major groups: rhabdomeric-type opsin (r-opsin) and ciliary-type opsin (c-opsin) [11,12,13]. In general, r-opsin is the main visual pigment in the rhabdomeres of insect compound eyes. Based on the spectral range to which opsins are sensitive, r-opsins of insects are classified into three categories: (1) long-wavelength-sensitive opsin (LW-opsin) with the peak absorbance more than 500 nm; (2) blue-sensitive opsin (BL-opsin) with the peak absorbance at 400–500 nm; and (3) ultraviolet-sensitive opsin (UV-opsin) with the peak absorbance at 300–400 nm [14,15,16]. *Drosophila* has 7 opsins (Rh1–7), of which Rh1, Rh2, and Rh5 are sensitive to blue light (absorption maxima of 486, 418, and 442 nm), Rh3 and Rh4 are sensitive to UV light (331 and 355 nm), and Rh6 is sensitive to green light (515 nm) [17,18,19,20]. Rh7 functions as a light detector and is involved in the regulation of the circadian rhythm [21]. It should be noted that different insect species vary in the number of opsins that they possess. For example, BL-opsin gene loss occurs in some species, such as *Tribolium castaneum* (red flour beetle, Coleoptera: Tenebrionidae), aphids (Aphidoidea), and planthoppers (Fulgoroidea) [22,23].

Opsins form covalent bonds with small molecular chromophores, thereby creating light-sensitive photopigments known as rhodopsins, which act as the primary activators in the phototransduction cascade [24]. The phototransduction pathway in *Drosophila* is most intensively studied. Light activates rhodopsin into metarhodopsin with chromophore conformational change, which initiates the G protein-mediated phototransduction cascade, leading to calcium and sodium ion exchange and the translation of light stimulus into an electrical signal [25,26,27]. A mutation in *LW-opsin* has been shown to alter gene expression in the phototransduction pathway in *Plutella xylostella* (diamondback moth, Lepidoptera: Plutellidae) [28]. Gene expression associated with the phototransduction pathway in the head of *Mythimna separata* (oriental armyworm, Lepidoptera: Noctuidae) was significantly altered following exposure to various light environments [29]. Thus, it was hypothesized that genes within the phototransduction pathway may enhance the phototaxis of the Asian citrus psyllid, making it essential to understand this pathway in *D. citri*.

Four visual photo-sensing opsins were identified in ACP: Dc-UV (UV-opsin), clustering with *Drosophila* Rh3 and Rh4; Dc-BW (BL-opsin), clustering with Rh5; Dc-LW (LW-opsin), clustering with Rh6; and Dc-UV-like, clustering with Rh7 [8]. The inhibition of *UV-opsin*, BL-opsin, and *LW-opsin* resulted in a reduction in phototactic response rates to the corresponding light source in ACP [8]. The expression of *LW-opsin* was observed to be the highest in ACP adults, in comparison to the other opsins [8]. Furthermore, only the expression of *LW-opsin* was found to be significantly up-regulated in response to white light treatment in comparison to dark treatment in our previous study (data not supplied). Accordingly, *LW-opsin* was selected as the target gene for further experimentation. To investigate the phototransduction pathway in *D. citri*, we first employed the RNA interference (RNAi) technique to inhibit *LW-opsin* gene expression, followed by RNA-Seq analysis to investigate the complete set of genes involved in phototransduction. Ultimately, inhibition of *LW-opsin* expression was observed to influence the mRNA expression levels of specific phototransduction genes, as determined by real-time quantitative PCR (RT-qPCR). This study advances our understanding of the phototransduction pathway in *D. citri* and provides theoretical guidance for identifying elicitors that could enhance *D. citri* phototaxis in the future.

## 2. Materials and Methods

### 2.1. Insects

The *D. citri* were reared on healthy *Murraya paniculata* within insect-rearing cages (60 × 40 × 75 cm) in an artificial greenhouse at the Institute of Plant Protection, Fujian Academy of Agricultural Sciences. The greenhouse was maintained under controlled conditions: temperature 26 ± 1 °C, relative humidity 60% ± 10%, and a 14:10 h (light:dark) photoperiod. To ensure a uniform age of the insects, newly molted adults were collected and placed in a cage (30 × 30 × 35 cm) with a plant for further experiments.

### 2.2. RNA Extraction and dsRNA Synthesis

The total RNA was extracted using the FreeZol reagent (Vazyme, Nanjing, China), purified with RNA Extraction Buffer (Beyotime, Shanghai, China), and precipitated with isopropanol. Then, 500 ng of total RNA was reverse transcribed into complementary DNA (cDNA) using the HiScript^®^ II 1st Strand cDNA Synthesis Kit (Vazyme), following the manufacturer’s protocol. The primers for the synthesis of double-stranded RNA (dsRNA) of *LW-opsin* and *EGFP* were designed according to Li et al. [8] and were synthesized by Sangon Biotech Co., Ltd. (Shanghai, China). The sequence fragment templates of *LW-opsin* and *EGFP* were amplified using the Phanta^®^ Super-Fidelity DNA Polymerase (Vazyme), according to the manufacturer’s protocol. The PCR products were purified using a FastPure^®^ Gel DNA Extraction Mini Kit (Vazyme), following the manufacturer’s protocol. A total of 2 μg of purified PCR product was employed for dsRNA synthesis in vitro using the T7 Ribomax™ Express large-scale production system (Promega, Madison, WI, USA), according to the manufacturer’s protocol.

### 2.3. RNA Interference of LW-Opsin

Injections of 200 ng of dsRNA were given to 10-day-old female *D. citri* adults using a Nanoject III microsyringe (Drummond Scientific Company, Broomall, PA, USA). After injection, the insects were reared on healthy *M. paniculata* in insect-rearing cages under the same conditions previously described. A total of 30 individuals were collected at 12, 24, 36, and 72 h post-injection, respectively. The dsLW-opsin and dsEGFP treatments were performed in triplicate at each of the four time points. Subsequently, the expression levels of *LW-opsin* were quantified via RT-qPCR to confirm the efficacy of the RNAi. The RT-qPCR amplification was conducted using the GoTaq^®^ qPCR Master Mix (Promega) with an initial step at 95 °C for 10 min, followed by 40 cycles at 95 °C for 15 s and 60 °C for 30 s. A final cycle was carried out at 95 °C for 15 s, 60 °C for 60 s, and 95 °C for 15 s. The gene *β-actin* of *D. citri* was employed as the reference gene. The primers for RT-qPCR were designed following Li et al. [8], and they were synthesized by Sangon Biotech Co.Ltd. (Shanghai, China). The comparative Ct method (2^−ΔΔCT^) was employed for the calculation of the transcript level [30].

### 2.4. Transcriptome Sequencing

RNA-Seq was conducted on samples collected at time points where *LW-opsin* expression levels were suppressed. The RNA integrity number (RIN) was assessed using an RNA 6000 Nano Lab Chip Kit and Bioanalyzer 2100 (Agilent Technologies, Santa Clara, CA, USA). Samples with an RIN value of 8.0 or higher were submitted to the Shanghai Meiji Biological Company (Shanghai, China) for library construction and sequencing using the Illumina Novaseq 6000 platform (San Diego, CA, USA). Adapter trimming and low-quality filtering of the raw reads were performed using fastp (https://github.com/OpenGene/fastp, accessed on 18 July 2024). The clean reads were then aligned to the *D. citri* genome (https://www.ncbi.nlm.nih.gov/datasets/genome/GCF_000475195.1/, accessed on 18 July 2024) using Bowtie2 (Version 2.4.1) and HISAT2 (http://ccb.jhu.edu/software/hisat2/index.shtml, accessed on 18 July 2024) for sequence analysis. The mapped reads were assembled and spliced with StringTie (http://ccb.jhu.edu/software/stringtie/, accessed on 18 July 2024) in accordance with the *D. citri* genome. New transcripts were identified by comparing them with known transcripts using the Gffcompare tool (Version 0.9.8).

### 2.5. Gene Function Annotation and Phototransduction Gene Enrichment

A BLASTx search was conducted in protein databases, including NR (non-redundant), Swiss-Prot, Pfam, EggNOG, GO (Gene Ontology), and KEGG (Kyoto Encyclopedia of Genes and Genomes), to determine functional annotation.

A comprehensive search of the transcriptome was performed with the keywords “phototransduction” to identify all genes related to the phototransduction pathway, based on the functional annotation of all protein databases described above. Subsequently, an enrichment analysis of the KEGG pathway was conducted via the Majorbio platform (https://www.majorbio.com/web/www/index, accessed on 31 July 2024). The annotation of the genes’ functions within the most enriched pathway was analyzed further.

### 2.6. Validation of Phototransduction Gene Expression by RT-qPCR

The objective was to validate whether the inhibition of *LW-opsin* expression affects the expression of genes involved in the phototransduction pathway. Accordingly, a subset of phototransduction genes was selected for further analysis. Primers for the selected genes are listed in Table 1. The RT-qPCR amplification was conducted following the previously described protocol.

### 2.7. Statistical Analysis

All statistical analyses were performed using the IBM SPSS software (version 22.0, IBM, Armonk, NY, USA). An independent *t*-test was used to assess significant differences in *LW-opsin* gene expression or phototransduction gene expression between the dsEGFP and dsLW-opsin treatment groups.

## 3. Results

### 3.1. RNA Interference of LW-Opsin

**Figure 1:**
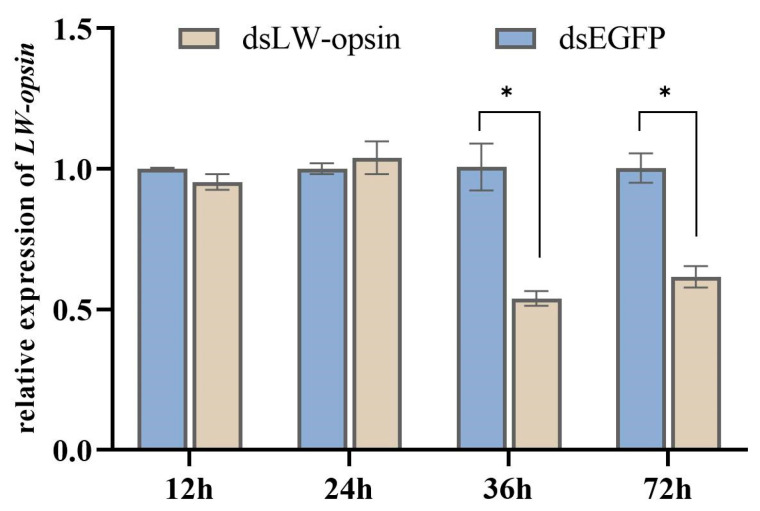
The interference efficiency of RNAi at the transcription level of *LW-opsin.* * indicates *p* < 0.05.

Following the injection of dsRNA (dsLW-opsin and dsEGFP), the relative expression levels of *LW-opsin* were quantified through RT-qPCR (Figure 1). The expression of *LW-opsin* had decreased by 46.82% at the 36 h time point and by 38.65% at the 72 h time point. Thus, the 36 h time point was considered optimal for conducting further RNA-Seq analysis.

### 3.2. Illumina Sequencing Analysis

A total of 40.97 Gb of clean data was obtained through transcriptome sequencing, with all samples exceeding 6.33 Gb of clean data. A total of 43,268 expressed genes were detected, comprising 23,604 known genes and 19,664 novel genes. In this paper, novel genes and novel transcripts are designated as “MSTRG”. Additionally, 83,232 transcripts were characterized, including 25,321 known transcripts and 57,911 novel transcripts. Approximately 74.38% of the high-quality clean data was retained for assembly and analysis. Furthermore, the percentage of *Q*_30_ bases exceeded 95.68% (Supplementary Data File S1), indicating a high level of base accuracy. The sequences of all transcripts were provided as supplementary material (Supplementary Data File S2).

### 3.3. Phototransduction Genes

A total of 87 genes, including 9 novel genes, were identified within the transcriptome as involved in phototransduction, based on KEGG functional annotation (Table 2). The 87 genes were assigned 19 distinct KO IDs. Of the 87 genes identified, 17 were associated with K08834 (MYO3, myosin III), 15 with K02183 (CALM, calmodulin), 10 with K04255 (Rh2_7, r-opsin), and 6 with K00910 (GRK, G-protein-dependent receptor kinase). Four genes were assigned to K04967 (TRPC4, transient receptor potential cation channel subfamily C member 4), four genes to K05858 (PLCB, phosphatidylinositol phospholipase C, beta), three genes to K02677 (PRKCA, classical protein kinase C alpha type), three genes to K04515 (CAMK2, calcium/calmodulin-dependent protein kinase II), and three genes to K04952 (CNGB1, cyclic nucleotide gated channel beta 1). Two genes belong to K04536 (GNB1, guanine nucleotide-binding protein G(I)/G(S)/G(T) subunit beta-1), two to K13803 (TRPL, transient-receptor-potential-like protein), two genes to K13806 (DAGL, sn1-specific diacylglycerol lipase), one to K04547 (GNG13, guanine nucleotide-binding protein G(I)/G(S)/G(O) subunit gamma-13), and one to K04634 (GNAQ, guanine nucleotide-binding protein G(q) subunit alpha). Additionally, one gene belongs to K04958 (ITPR1, inositol 1,4,5-triphosphate receptor type 1), one to K07972 (GNB, guanine nucleotide-binding protein subunit beta), one to K12322 (GUCY2F, guanylate cyclase 2F), and one to K13805 (ARR2, arrestin2).

### 3.4. KEGG Pathway Enrichment of Phototransduction Genes

**Figure 2:**
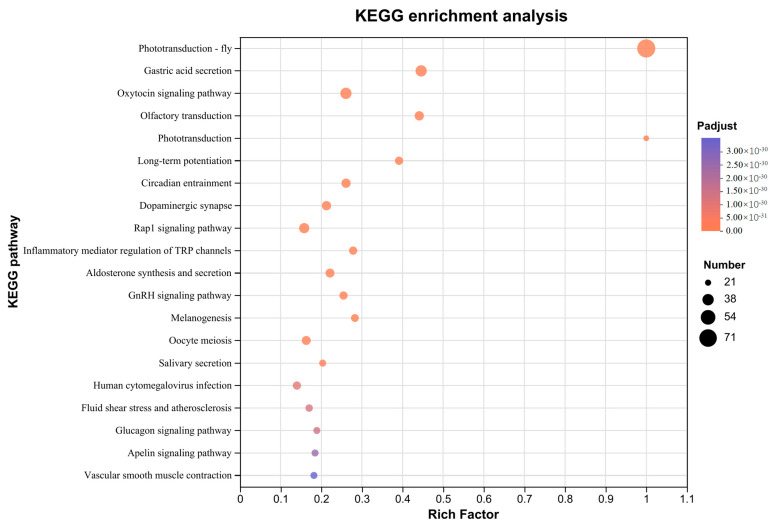
Top 20 of KEGG pathway enrichment of phototransduction genes.

The phototransduction-fly (map04745, rich factor = 1), phototransduction (map04744, rich factor = 1), and circadian entrainment (map04713, rich factor = 0.26) pathways, which are associated with light perception, were significantly enriched (Figure 2, Supplementary Data File S3). A total of 71 genes were enriched in the phototransduction-fly pathway, 21 genes were enriched in the phototransduction pathway, and 30 genes were enriched in the circadian entrainment pathway.

### 3.5. Gene Function Annotation Based on Phototransduction-Fly Pathway

**Figure 3:**
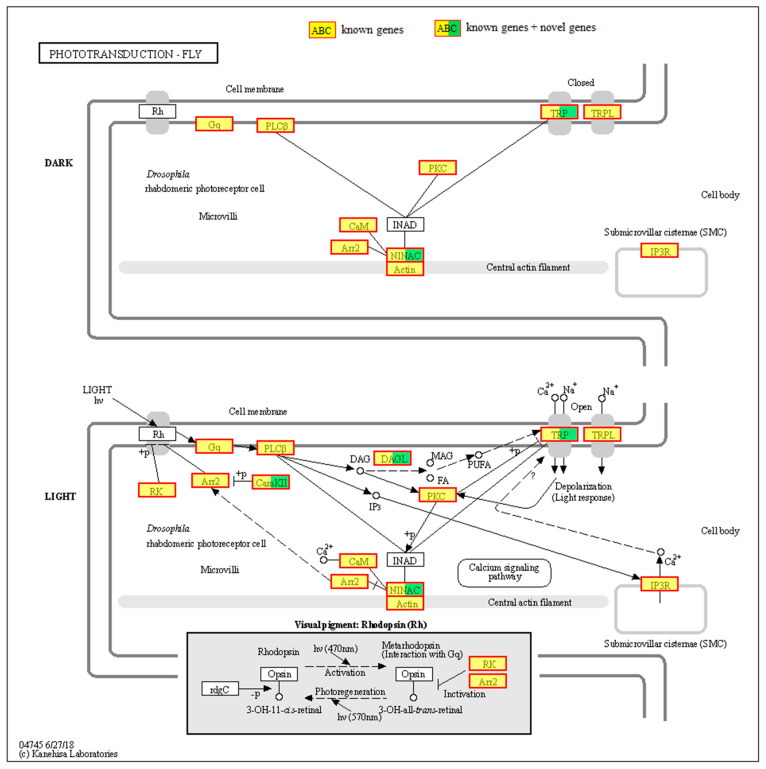
Gene function annotation based on phototransduction-fly pathway. Rh, rhodopsin. Gq, heterotrimeric Gq protein. PLCβ, phospholipase C β. TRP, transient receptor potential. TRPL, transient receptor potential like. PKC, protein kinase C. CaM, calmodulin. Arr2, arrestin2. NINAC, neither inactivation nor afterpotential C. IP3R, inositol 1,4,5-trisphosphate. DAGL, diacylglycerol lipase. CaMKII, CaM kinase II. RK, G protein-coupled receptor kinase 1.

A total of 71 genes were found to function in accordance with the phototransduction-fly pathway, as illustrated in Figure 3 and detailed in Table 3. The genes *LOC103516584*, *LOC103513214*, and *LOC103507998* were identified as encoding the α-, β-, and γ-subunits of the heterotrimeric Gq protein, respectively. Four genes were identified as encoding phospholipase C β (PLCβ), four as encoding the cation channel TRP (transient receptor potential), and two as encoding the cation channel TRPL (transient receptor potential like). Three genes were identified as encoding protein kinase C (PKC), fifteen as encoding CaM (calmodulin), and one as encoding Arr2 (arrestin2). Seventeen genes were identified as encoding NINAC (neither inactivation nor afterpotential C), ten as encoding actin, and one as encoding IP3R (inositol 1,4,5-trisphosphate, InsP3). Two genes were identified as encoding DAGL (diacylglycerol lipase), three as encoding CaMKII (CaM kinase II), and six as encoding RK (G protein-coupled receptor kinase 1). To confirm the precise details of these genes, further phylogenetic analysis, gene cloning, and characterization will be required.

It is noteworthy that ten genes (*LOC103506067*, *LOC103506845*, *LOC103516743*, *LOC103516911*, *LOC103516912*, *LOC103517452*, *LOC103517454*, *LOC103521071*, *LOC103524061*, *LOC113473058*) classified within the seven-transmembrane G protein-coupled receptor superfamily were not identified as opsins. Additionally, INAD (inactivation nor afterpotential D) and rdgC (retinal degeneration C) were not identified in our transcriptome.

### 3.6. Knockdown of LW-Opsin Gene Changes the Gene Expression of the Phototransduction Pathway

**Figure 4:**
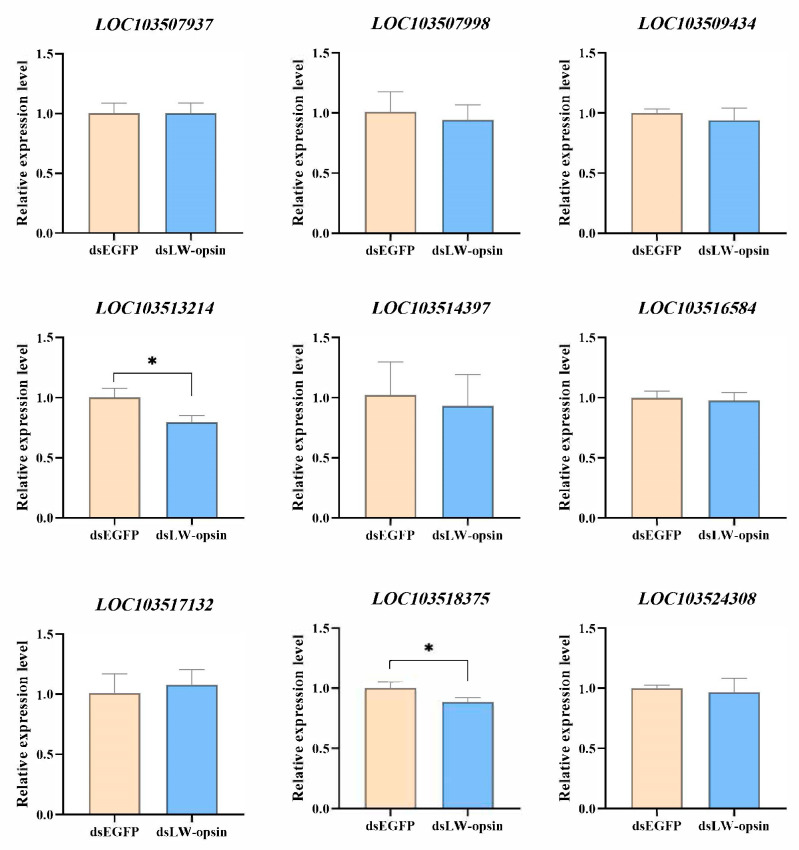
Verification of the 9 phototransduction genes whose expression is influenced by the knockdown of *LW-opsin* gene expression. * indicates *p* < 0.05.

A total of nine genes were selected for validation to determine whether the knockdown of the *LW-opsin* gene alters the expression of genes involved in the phototransduction pathway. The results demonstrated that only the *LOC103513214* (*Gqβ*) and *LOC103518375* (*ninaC*) genes exhibited a reduction in expression when *LW-opsin* gene expression was inhibited (Figure 4).

## 4. Discussion

The *Drosophila* phototransduction pathway (phototransduction-fly) has been extensively studied [13,24,25,26,27]. In this study, a total of 71 genes in *D. citri* were identified as enriched in the phototransduction-fly pathway, including *Gqα*, *Gqβ*, *Gqγ*, *Arr2*, *PLCβ*, *TRP*, and *TRPL*, among others, with the exception of *INAD* and *rdgC*. This suggests that the phototransduction pathway in *D. citri* is comparable to that observed in *Drosophila*, although potential variations cannot be ruled out. Furthermore, the phototransduction pathway in non-*Drosophila* insects has been investigated through transcriptome-sequencing techniques. For example, the majority of phototransduction genes were found to be conserved between *D. melanogaster* and Lepidoptera, including *Gqα*, *Gqβ*, *Gqγ*, *Arr1*, *Arr2*, *trp*, *trpl*, and others [31]. In *Ptomaphagus hirtus* (troglobiont beetle, Coleoptera: Leiodidae), approximately 20 genes have been identified, including *LW-opsin*, *arr1*, *arr2*, *trp*, and *trp* [32]. Moreover, studies have demonstrated that gene expression in the phototransduction pathway is altered by environmental stimuli. For instance, eight genes, including *arr2*, *BRh*, and *rdgC*, have been demonstrated to exhibit seasonal adaptations in expression in *Bicyclus anynana* (small brown butterfly, Lepidoptera: Nymphalidae) [33]. Gene expression levels in the phototransduction pathway, including *Gq*, *PLCβ*, *PKC*, *TRP*, *TRPL*, *INAD*, *Arr2*, *NINAC,* and *CamkII*, change when *M. separata* is exposed to different light environments [29]. These studies suggest that the phototransduction pathway is relatively conserved in insects. Furthermore, the expression of the phototransduction genes may be altered in response to changes in the external environment, particularly light.

The functions of phototransduction genes observed in *D. citri* have been well-demonstrated in *Drosophila*. For example, mutations in the α-, β-, or γ-subunits of the heterotrimeric Gq protein led to reduced light sensitivity [34,35,36]. Arr2 is a key inhibitory protein responsible for rhodopsin inactivation, which subsequently causes photoreceptor cell inactivation [37]. IP3R, an InsP_3_ receptor that regulates calcium channels, is linked to retinal degeneration [38,39]. NINAC includes two isoforms, P132 and P174, both of which have a protein kinase structural domain that can bind the myosin head structural domain, but the isoforms have different C-termini, both isoforms are involved in regulating the transport of CaM and Arr2, with P174 mutations resulting in brief reactivation of the photoresponse even after light stimulation ceases [40,41,42]. In the future, more physiological, behavioural, and mechanistic studies are required to gain a comprehensive understanding of the phototransduction pathway in *D. citri*. With the increasing accessibility of molecular technologies, such as CRISPR/Cas9-based genome editing technology and RNAi technology, research on the function of phototransduction genes in *D. citri* will be a promising area for future exploration.

The functional relationship between opsins and phototactic behavior is firmly established [8,43,44,45,46]. Although opsins serve as the initial stage in the phototransduction pathway, few studies have explored changes in downstream genes following opsin gene knockout or knockdown. A recent study has demonstrated that the *LW-opsin* mutation affects the expression of genes involved in the phototransduction pathway in *P. xylostella*, including *NINAC*, *rdgC*, *TRP*, *TRPL*, *INAD*, and others [28]. In our study, only two of the nine validated genes exhibited down-regulated gene expression following the knockdown of the *LW-opsin* gene. This may be due to the fact that entire female bodies were used as samples in our study, as opposed to only the heads used in previous studies [28,29,31,32,33]. Further validation is required to confirm whether the expression of genes involved in the phototransduction pathway is affected when *LW-opsin* expression is suppressed using only the heads of *D. citri*.

## 5. Conclusions

In conclusion, a total of 71 genes in *D. citri* were identified as enriched in the phototransduction-fly pathway. These genes encode key proteins in this process, including *Gqα*, *Gqβ*, *Gqγ*, *PLCβ*, *TRP*, and *TRPL*, among others. Moreover, the *LOC103513214* and *LOC103518375* genes exhibited a reduction in expression when *LW-opsin* gene expression was suppressed. Our results contribute to a deeper understanding of the molecular mechanisms underlying *D. citri* vision. This knowledge will facilitate the identification of elicitors that could enhance the understanding of *D. citri* phototaxis moving forward.

## Figures and Tables

**Table 1:** Primers used to validate the expression of phototransduction genes affected by inhibition of *LW-opsin* expression.

Gene ID in NCBI	Homolog Name	Forward Primer Sequence (5′ to 3′)	Reverse Primer Sequence (5′ to 3′)
LOC103507937	*IP3R*	aggcctcgtcagaaagaata	gagctcagcacacttttaca
LOC103507998	*Gq*γ	tccatcatgtaactacccgt	tgttcaggtgacgatcattg
LOC103509434	*PLCβ*	tactcacgggaaagctatgt	cgttttgcagcagtgatttt
LOC103513214	*Gq*β	agggagaagaaatgtgtcca	ctatctgttgatctgcacgg
LOC103514397	*RK*	caatgaacgacttctccgtt	cattttaccggtatccgctt
LOC103516584	*Gq*α	catatccttggttccagcac	tttgacagcgcagaatacaa
LOC103517132	*PKC*	cagatcggcaaattcaagga	aaatggaggttgtcccacta
LOC103518375	*NINAC*	aagagaggaagacatcaggg	gggcaaatctccttgttgat
LOC103524308	*Arr2*	cgctctgaagaagtcgaaaa	cagttgtttattcgtccggt

**Table 2:** Genes involved in phototransduction identified through KEGG functional annotation.

KO ID	KO Name	Gene IDs	Gene Number
K00910	GRK	LOC103505361, LOC103505365, LOC103519341, LOC103523400, LOC103524942, LOC103514397	6
K02183	CALM	LOC103505048, LOC103509805, LOC103509868, LOC103510651, LOC103511026, LOC103511569, LOC103511781, LOC103512666, LOC103513726, LOC103514274, LOC103517588, LOC103521078, LOC113468030, LOC103507684, LOC113465625	15
K02677	PRKCA	LOC108253019, LOC108253020, LOC103517132	3
K04255	Rh2_7	LOC103506067, LOC103506845, LOC103516743, LOC103516911, LOC103516912, LOC103517452, LOC103517454, LOC103521071, LOC103524061, LOC113473058	10
K04515	CAMK2	LOC103509280, LOC113471771, MSTRG.19456	3
K04536	GNB1	LOC103515883, LOC103515885	2
K04547	GNG13	LOC103507998	1
K04634	GNAQ	LOC103516584	1
K04952	CNGB1	LOC103517837, LOC103522372, LOC103520508	3
K04958	ITPR1	LOC103507937	1
K04967	TRPC4	LOC113472687, LOC108252237, LOC108252236, MSTRG.12236	4
K05858	PLCB	LOC103508056, LOC103513753, LOC103519973, LOC103509434	4
K06636	SMC1	LOC113465618, LOC113466617, LOC113466908, LOC113467623, LOC113467958, LOC113467961, LOC113470296, LOC113473712, LOC113473717, LOC113473739	10
K07972	GNB	LOC103513214	1
K08834	MYO3	LOC103509649, LOC103513890, LOC103514755, LOC103515283, LOC103515285, LOC103515286, LOC103517045, LOC103517041, LOC103517042, LOC103517043, LOC103518375, MSTRG.45354, MSTRG.43624, MSTRG.48618, MSTRG.5326, MSTRG.17197, MSTRG.35405	17
K12322	GUCY2F	LOC103508103	1
K13803	TRPL	LOC103508643, LOC103522058	2
K13805	ARR2	LOC103524308	1
K13806	DAGL	LOC103515251, MSTRG.11676	2

**Table 3:** Genes associated with the phototransduction-fly pathway.

Gene Name	Gene IDs	Gene Number
*Gq*	LOC103516584, LOC103513214, LOC103507998	3
*PLCβ*	LOC103519973, LOC103509434, LOC103513753, LOC103508056	4
*TRP*	LOC113472687, LOC108252236, LOC108252237, MSTRG.12236	4
*TRPL*	LOC103508643, LOC103522058	2
*PKC*	LOC103517132, LOC108253019, LOC108253020	3
*CaM*	LOC103505048, LOC103509805, LOC103509868, LOC103510651, LOC103511026, LOC103511569, LOC103511781, LOC103512666, LOC103513726, LOC103514274, LOC103517588, LOC103521078, LOC113468030, LOC103507684, LOC113465625	15
*Arr2*	LOC103524308	1
*NINAC*	LOC103509649, LOC103513890, LOC103514755, LOC103515283, LOC103515285, LOC103515286, LOC103517045, LOC103517041, LOC103517042, LOC103517043, LOC103518375, MSTRG.5326, MSTRG.17197, MSTRG.35405, MSTRG.43624, MSTRG.45354, MSTRG.48618	17
*Actin*	LOC113465618, LOC113466617, LOC113466908, LOC113467623, LOC113467958, LOC113467961, LOC113470296, LOC113473712, LOC113473717, LOC113473739	10
*IP3R*	LOC103507937	1
*DAGL*	LOC103515251, MSTRG.11676	2
*CamK* *II*	LOC113471771, LOC103509280, MSTRG.19456	3
*RK*	LOC103505361, LOC103505365, LOC103519341, LOC103523400, LOC103524942, LOC103514397	6

## Data Availability

All data are available in publicly accessible repositories (https://dataview.ncbi.nlm.nih.gov/object/PRJNA1178351, accessed on 1 October 2024, Accession: PRJNA1178351).

## Supplementary Materials

The following supporting information can be downloaded at https://www.mdpi.com/article/10.3390/insects15120966/s1, Supplementary Data File S1: Summary of transcriptome-sequencing results generated from *Diaphorina citri*; Supplementary Data File S2: The sequences of all transcripts; Supplementary Data File S3: KEGG pathway enrichment of phototransduction genes.
