# Supplementary material for: Transcriptomic Characterization of Phototransduction Genes of the Asian Citrus Psyllid Diaphorina citri Kuwayama
Source: Insects. 2024 Dec 4;15(12):966. doi: 10.3390/insects15120966 (PMC11678440; doi:10.3390/insects15120966)
Supplement: Supplementary file 1 [file insects-15-00966-s001.zip › Supplementary Data File 1-Summary of transcriptome-sequencing results generated from Diaphorina citri.pdf]

Table S1. Summary of transcriptome-sequencing results generated from *Diaphorina citri*

**Sample**	Q_{30}	Total mapped	Multiple mapped	Uniquely Mapped
EGFP 1	96.18%	34,469,705 (74.9%)	6,759,118 (14.69%)	27,710,587 (60.21%)
EGFP 2	96.13%	35,924,269(74.76%)	6,897,063 (14.35%)	29,027,206 (60.41%)
EGFP 3	96.14%	33,647,543(74.83%)	6,235,894 (13.87%)	27,411,649 (60.96%)
LW 1	95.68%	31,540,824(74.39%)	5,823,764 (13.74%)	25,717,060 (60.66%)
LW 2	96.10%	36,847,165(74.75%)	6,958,250 (14.12%)	29,888,915 (60.64%)
LW 3	95.82%	32,240,800(74.38%)	5,795,174 (13.37%)	26,445,626 (61.01%)
